# Asporin Interacts With HER2 to Promote Thyroid Cancer Metastasis *via* the MAPK/EMT Signaling Pathway

**DOI:** 10.3389/fonc.2022.762180

**Published:** 2022-05-02

**Authors:** Shaohua Zhan, Tianxiao Wang, Jingying Li, Hanyang Zhu, Wei Ge, Jinming Li

**Affiliations:** ^1^National Center for Clinical Laboratories, Institute of Geriatric Medicine, Chinese Academy of Medical Sciences, Beijing Hospital/National Center of Gerontology, Beijing, China; ^2^Institute of Basic Medical Sciences, State Key Laboratory of Medical Molecular Biology & Department of Immunology, Chinese Academy of Medical Sciences, Beijing, China; ^3^Department of Laboratory Medicine, Peking University Third Hospital, Beijing, China; ^4^Key Laboratory of Carcinogenesis and Translational Research, Department of Head and Neck Surgery, Peking University Cancer Hospital & Institute, Beijing, China

**Keywords:** papillary thyroid cancer, asporin, HER2, quantitative proteomics, tumor migration and invasiveness

## Abstract

Approximately 85% of histological subtypes of thyroid cancer are papillary thyroid cancer (PTC), and the morbidity and mortality of PTC patients rapidly increased due to lymph node metastases or distant metastasis. Therefore, it needs to distill an enhanced understanding of the pathogenesis of PTC patients with lymph node metastases or distant metastasis. We employed the TMT-based quantitative proteomics approach to identify and analyze differentially expressed proteins in PTC with different degrees of lymph node metastases. Compared with paired normal tissues, asporin is overexpressed in PTC-N0, PTC-N1a, and PTC-N1b tumorous tissues *via* proteomics, western blotting, and immunohistochemistry assays. Functionally, asporin is mainly expressed in the extracellular matrix, cell membrane, and cytoplasm of PTC tumorous tissues, and promotes thyroid cancer cell proliferation, migration, and invasion. Mechanistically, asporin, interacting with HER2, co-localizes HER2 on the cell membrane and cytoplasm, and the asporin/HER2/SRC/EGFR axis upregulate the expression of EMT-activating transcription factors through the MAPK signaling pathway. Clinically, asporin can be regarded as a serological biomarker to identify PTC patients with or without lymph node metastasis, and high expression of asporin in PTC tumorous tissues is a risk factor for poor prognosis.

## Introduction

Cancer has rapidly grown in global incidence and mortality in recent years and is expected to become the leading cause of death from the non-communicable disease according to GLOBOCAN (1). Thyroid cancer is the most common endocrine malignancy, and its incidence was the highest among all cancers in the United States between 2000 and 2009 (2). Approximately 85% of histological subtypes of thyroid cancer are papillary thyroid cancer (PTC). Although the death rate of PTC is relatively low following surgery with or without concomitant radioiodine treatment, the morbidity and mortality of PTC patients are increased greatly due to lymph node metastases (LNMs) or distant metastasis (3).

PTC is MAPK-driven cancer characterized by mutually exclusive drivers including *BRAF^V600E^* and mutated *RAS* (4). Importantly, the BRAF V600E oncoprotein (encoded by the *BRAF^V600E^* mutation) is a typical member of MAPK signaling pathway, occurring in 40%–60% of PTC patients (3). Furthermore, Xing M demonstrated that activation of MAPK signaling pathway resulted in upregulation of tumor-promoting genes (e.g. *VEGFA*, *MET*, *HIF1A, UPA*, *UPAR*, *TGFB1*, and *TSP1*) as well as downregulation of tumor suppression and thyroid genes (e.g. *TIMP3*, *SLC5A8*, *DAPK1*, *NIS, TSHR*, and *TPO*) *(* 2). Recently, the framework of *BRAF^V600E^*-*RAS* gene expression scores in The Cancer Genome Atlas (TCGA) indicated that PTCs differentiate into *BRAF^V600E^*-like and *RAS*-like PTCs (5). All these studies indicated that the MAPK signaling pathway (upregulated in tumors with the *BRAF^V600E^* mutation) is associated with PTC aggressiveness. However, the phase 2 trial of vemurafenib targeting BRAF-mutated PTC patients showed only a 38.5% response rate, which is considerably lower than that in patients with BRAF-mutated melanoma (6). Therefore, it needs to distill an enhanced understanding of the pathogenesis of PTC patients.

Asporin belongs to the class I small leucine-rich proteoglycan (SLRP) family, which also includes biglycan and decorin (7). The name “asporin” refers to its unique aspartate resides (D-repeat) in its N-terminal domain and its 54% identity with the sequence of decorin (8). Asporin contains 380 amino acids and its D-repeat polymorphisms (residues 8–19) in the N-terminus are correlated with osteoarthritis and metastatic recurrence of prostate cancer (7, 9). Although decorin acts as a tumor suppressor and biglycan is regarded as an oncogene, asporin exerts tumor-suppressor function in triple-negative breast cancer but exerts tumor-promotor function in some types of cancer, including breast, pancreatic, colorectal, gastric, and prostate cancer (10). For example, asporin binds directly to extracellular TGF-β1 in triple-negative breast cancer and its downstream cytoplasmatic component Smad 2/3 in colorectal cancer, resulting in inhibition or activation of the TGF-β1 signaling pathway, respectively (11, 12). Therefore, it is unsurprising that asporin plays different roles depending on binding different proteins. A growing body of evidence now demonstrates that asporin acts as an extracellular matrix component or intracellular protein that positively or negatively controls proliferation, invasion, and metastasis of cancer cells by regulating the TGF-β, EGFR, and CD44 signaling pathways (10). A previous study indicated that asporin is expressed at moderate levels in thyroid normal tissues (8); however, the biological roles of asporin in thyroid cancer progression have never been investigated. The present study contributes to this field by demonstrating that asporin interacts with HER2 to promote thyroid cancer metastasis by regulating the MAPK-epithelial-to-mesenchymal transition (EMT) axis.

## Materials and Methods

### Patients and Specimens

In total, 106 PTC patients were recruited from the department of head and neck surgery, Peking University Cancer Hospital & Institute, People’s Republic of China. The use of human materials in this study was approved by the Ethics Committee of Peking University Cancer Hospital & Institute and informed consent was obtained from all patients. Tumorous and paired normal tissues from 53 PTC patients were used in Western blotting, immunohistochemistry (IHC), and tandem mass tag (TMT)-based mass spectrometry (MS)/MS assays. Serum samples from another 53 PTC patients were analyzed by ELISA. Tissues collected from surgical procedures and serum samples were immediately snap-frozen in dry ice and then stored at –80°C. The clinicopathological parameters of all these PTC patients are summarized in **Supplementary Table 1**.

### Co-Immunoprecipitation

Immunoprecipitation was performed as previously described (13, 14). Briefly, whole-cell extracts were obtained with RIPA buffer (ab156034; Abcam) containing 1 mM PMSF and a protease inhibitor cocktail (04693132001; Roche). After centrifugation at 12,000 ×*g* for 20 min at 4°C, soluble proteins were quantified by BCA. Samples (1 mg) of proteins precleared with 30 μl protein A/G Plus-Agarose (sc-2003; Santa Cruz) were used for each immunoprecipitation experiment. Proteins were incubated with 2 μg antibodies and 30 μl protein A/G Plus-Agarose. Immunoprecipitated materials were washed four times with ice-cold wash buffer (0.1% Triton X-100, 50 mM Tris-HCl, pH 7.4, 300 mM NaCl, 5 mM EDTA, 0.02% sodium azide) and once more using 1 ml ice-cold PBS. Bound proteins were separated by SDS-PAGE, transferred onto PVDF membranes, and immunoblotted with the appropriate antibodies. Signals were detected with Enhanced Chemiluminescence kits (Millipore) according to the manufacturer’s instructions. Band intensity was measured using FluorChem Q 3.4.0 software.

### IHC and Evaluation of Staining

Tissue sections (5 μm thick) were de-waxed at 60°C for 30 min followed by two 5-min washes with xylene. The sections were then rehydrated by sequential 5-min washes in 100%, 95%, and 80% ethanol and distilled water. Antigen retrieval was performed by heating the tissues at 95°C for 10 min in 0.01 M citrate buffer (pH 6.0). The endogenous peroxidase activity of the tissues was blocked by 3% hydrogen peroxide for 30 min, followed by incubation with primary detection antibodies overnight at 4°C. The sections were then incubated with the Polink-2 Plus^®^ HRP Polymer Detection System (PV-9001 and PV-9002; ZSGB-BIO) according to the manufacturer’s instructions. The samples were developed using the 3, 3′-diaminobenzidine (DAB) substrate (Dako), and counterstained with hematoxylin.

The immunohistochemical staining was evaluated according to percentage and intensity. The percentage of positive cells was scored as 0–4 (0 = <10% cells; 1 = 10%–30% cells, 2 = 30%–50% cells, 3 = 50%–70% cells, and 4 = >70% cells), and the staining intensity of the positive cells was scored as 0–3 (0 = no staining, 1 = weak staining, 2 = intermediate staining, and 3 = strong staining). The percentage and intensity scores were summed to obtain the final immunohistochemical staining scores ranging from 0–7. Based on these scores, the protein expression level was classified into three groups: 0–2 = negative staining; 3–5 = moderate staining; and 6–7 = high staining.

### Immunofluorescence Staining

Immunofluorescence staining was performed as previously described (15). Briefly, BCPAP and KTC-1 cells were washed three times with PBS, fixed in 4% paraformaldehyde for 20 min, permeabilized with 0.2% Triton-X 100 for 15 min, and then blocked with 5% BSA for 60 min. Cells were incubated with primary detection antibodies (anti-asporin and anti-HER2 (sc-7301); Santa Cruz) at 4°C overnight. Cells were then incubated with appropriate secondary detection antibodies [Alexa Fluor Plus 555-conjugated anti-mouse (A32727; Thermo Fisher) or anti-rabbit (A32727) antibody and Alexa Fluor 488-conjugated anti-rabbit (A11034; Thermo Fisher)]. Cell nuclei were stained with DAPI (Sigma) at a final concentration of 0.1 mg/mL. Fluorescent images were captured on a laser confocal microscope (LSM780; ZEISS).

### Transfection of Thyroid Cancer Cell Lines

Poorly differentiated thyroid cancer cell lines [B-CPAP (RRID: CVCL_0153) and KTC-1 (RRID: CVCL_6300)] and anaplastic thyroid cancer (ATC) cell lines [BHT-101 (RRID: CVCL_1085)] were kindly provided by the Stem Cell Bank of the Chinese Academy of Sciences. BCPAP and KTC-1 cells were cultured in RPMI 1640 supplemented with 10% FBS and 1% non-essential amino acids (Invitrogen, USA). BHT101 cells were cultured in DMEM supplemented with 20% FBS. All human cell lines have been authenticated using short tandem repeat profiling within this year, and all experiments were performed with mycoplasma-free cells. Small interfering RNAs (siRNAs) were obtained from Guangzhou RiboBio (China). Three siRNAs targeting the *Asporin* gene were designed and synthesized (siRNA2: 5’-GTGACGGTGTTCCATATCA-3’; siRNA4: 5’-GGAGTATGTGCTCCTATTA-3’; siRNA5: 5’-GTGCTATTCACGAGTTGTA-3’). At the time of transfection, cells were plated onto a 6-well plate at 60%–80% confluence. Transfection was performed with RNAiMAX (13778-150; Thermo Fisher) according to the manufacturer’s protocol. RNAiMAX reagent (7.5 μL) and siRNAs were diluted in Opti-MEM and incubated at room temperature for 15 min. The mixtures were then added to cells, giving a final concentration of siRNAs of 30 pmol. BCPAP, KTC-1, and BHT101 cells were cultured for 72 h after transfection and were subsequently lysed in RIPA buffer (ab156034; Abcam).

### Drug Treatment

For the Afatinib assay, BCPAP and KTC-1 Cells (4×10^5^) were incubated with 100 ng/ml EGF (236-EG-200; R&D Systems) for 20 min, and then incubated with or without 1 μmol/L Afatinib (S1011; Selleckchem) for 2 h. For the PLX4032 assay, BCPAP and KTC-1 Cells (4×10^5^) were also incubated with 100 ng/ml EGF for 20 min and then incubated with or without 2 μmol/L PLX4032 (S1267; Selleckchem) for 4 h. After Afatinib and PLX4032 treatment, BCPAP and KTC-1 Cells were washed with ice-cold PBS three times, and whole-cell lysates were subjected to SDS–PAGE and incubated with p-EGFR^Y845^, p-ERK1/2, t-ERK1/2, SLUG, ZEB1, ZEB2, and β-actin antibodies, respectively.

### *In Vitro* Assays of Cell Migration and Invasion

The migratory and invasive potentials of the BCPAP, KTC-1 and BHT101 cell lines were evaluated as described previously (16). Briefly, 3×10^4^ cells suspended in RPMI 1640 or DMEM media were seeded in the upper chamber of the Transwell (3422; Corning) coated with 100 μl 2% Matrigel (356234; Corning). RPMI 1640 or DMEM supplemented with 10% FBS was placed in the lower chamber as the source of chemoattractant. After 24 h of 37°C incubation, the cells remaining on the upper surface of the insert were removed using a cotton swab, and the cells on the lower surface were fixed with anhydrous methanol for 30 min and then stained with 0.2% crystal violet solution (V5265-250ML; Sigma). For each insert, cells in the center and five randomly selected peripheral fields were assessed under an inverted microscope. Migration assays were performed using the invasion assay method, except that 5×10^4^ cells were seeded into the upper chamber that was not coated with Matrigel.

### Cell Proliferation And Colony Formation Assays

Cell proliferation assays were performed using the Cell Counting Kit-8 (96992; Sigma) according to the manufacturer’s protocol. Briefly, 5×10^3^ BCPAP and KTC-1 cells in suspension were seeded into a 96-well plate (100 μl/well). After incubating the plate in a humidified incubator (37°C, 5% CO_2_) for 24, 48, 72, and 96 h, 10 μL CCK-8 solution was added to each well. After incubating the plate for 2 h, the absorbance values at 450 nm and 600 nm were measured using a microplate reader (Multiskan FC; Thermo Scientific). For the colony formation assay, 3×10^3^ BCPAP and KTC-1 cells suspended in RPMI 1640 containing 10% FBS were added to each well of a 6-well plate. Cells were cultured for 14 days at 37°C, and colonies were counted in three independent experiments.

### TMT-Based MS/MS Analysis and Protein Identification

TMT-based MS/MS analysis was performed as previously described (17, 18). Briefly, according to the lymph node status, 48 thyroid tissues from 24 PTC patients were pooled as follows: tumorous tissues from PTC patients with N0 (N0_T), tumorous tissues from PTC patients with N1a (N1a_T), tumorous tissues from PTC patients with N1b (N1b_T), and paired normal tissues from all PTC patients (N0_N, N1a_N, and N1b_N). The four groups of proteins were reduced by incubation with 10 mM DTT for 30 min at 55°C, alkylated with 25 mm IAA for 30 min at room temperature in the dark, and then incubated with trypsin/Lys-C mix at a protein/protease ratio of 25:1 for 12 h at 37°C. Subsequently, TMT isobaric label reagents (0.8 mg TMT dissolved in 40 μL 99.9% acetonitrile) were used separately according to the manufacturer’s instructions to label each group of peptides as follows: TMT-126 for N1b_T; TMT-127 for N0_T; TMT-128 for N1a_T; TMT-131 for N0+N1a+N1b_N. All the labeled peptides in the four groups were then combined for subsequent high-performance liquid chromatography (HPLC) and LC-MS/MS analysis (18).

The MS/MS raw data were analyzed against the human reviewed Swiss-Prot FASTA database (released on 2018.03.02) using Proteome Discoverer software (Version 2.1, Thermo Scientific). The following search criteria were applied: carbamidomethylation (C, +57.021 Da) and TMT-6plex (K and peptide N-terminus) as fixed modifications and oxidation (methionine, M) as a variable modification. A maximum of two missed trypsin/Lys-C cleavages was allowed. The false discovery rate (FDR) was determined based on searches of the peptide spectrum matched against the reversed decoy database. The FDRs for peptide and protein identification were both set to 0.01. The MS/MS raw data were deposited in the ProteomeXchange Consortium *via* the PRIDE partner repository with the dataset identifier PXD007971.

### Bioinformatic Analysis

The two-sided 95% prediction interval of the combined ratio distribution was used to identify the cutoffs for differentially expressed proteins (DEPs) (set as ≥1.7-fold or ≤0.4-fold) using JMP Pro 13.2.1 software (**Supplementary Figure 1A**). Gene ontology (GO) and pathway enrichment analyses were performed using the Funrich tool (Version 3.1.3). The protein-protein interaction (PPI) analysis was performed and visualized using the stringAPP plugin in Cytoscape (Version 3.7.0), with a confidence cutoff set at 0.4. TCGA-Assembler 2 software was used to download the normalized RNA-seq by expectation-maximization (RSEM) data and clinicopathological parameters of thyroid cancer (THCA) from TCGA (19). Upregulated mRNA expression was defined as a Z-score ≥1, whereas downregulated mRNA expression was defined as a Z-score ≤−1 according to the previous study (20).

### Statistical Analysis

All statistical analyses were performed using SPSS 19.0 (IBM Corp., Armonk, NY, USA) and JMP Pro 13.2.1 software. Each experiment was repeated at least 3 times. ANOVA was used to evaluate differences among different groups and Tukey’s HSD was further applied for pairwise comparisons. Mann–Whitney or Kruskal–Wallis tests were used to analyze the relationship between *asporin* mRNA expression and clinicopathological characteristics. The Kaplan–Meier method was used to evaluate progression-free survival (PFS) and overall survival (OS). Receiver operating characteristic (ROC) curves were generated to evaluate the diagnostic value of serum asporin in thyroid cancer. Two-tailed *P* < 0.05 was considered to indicate statistical significance.

## Results

### Overview of Proteomic Profiles and Corresponding Bioinformatic Analysis

**Figure 1:**
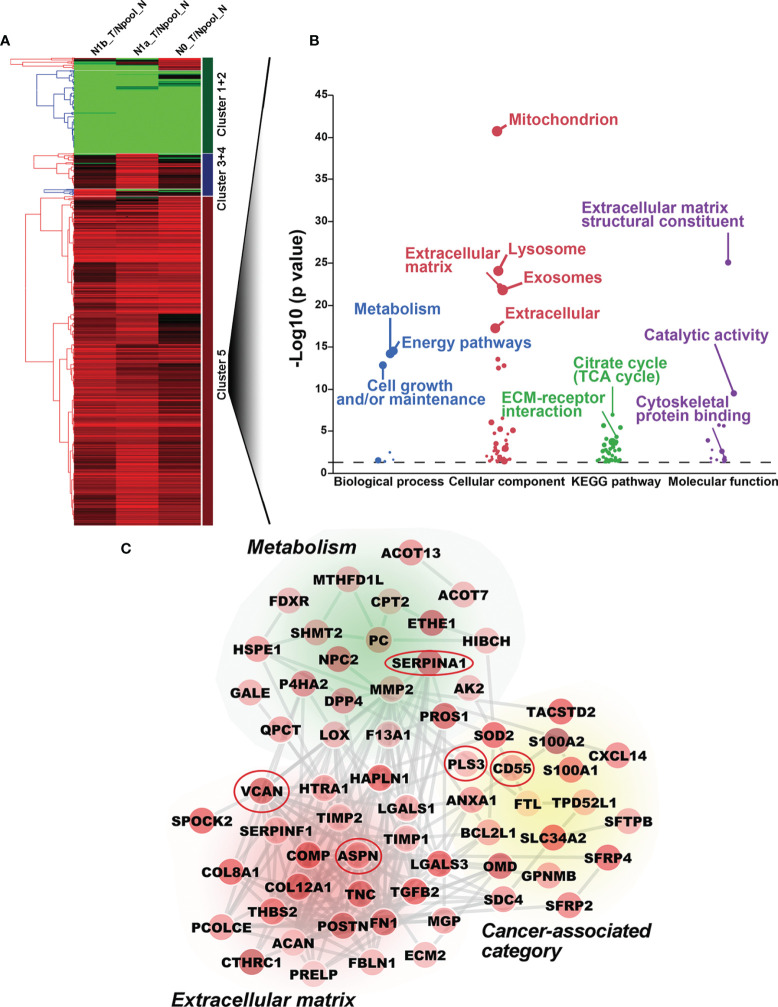
Bioinformatic analysis of differentially expressed proteins (DEPs) by TMT-based MS/MS. **(A)** Hierarchical cluster analysis of DEPs. Upregulated and downregulated DEPs are shown in red and green, respectively. Five clusters are shown on the left of the heatmap. **(B)** Gene ontology and pathway enrichment analysis of DEPs in Cluster 5. The biological process, cellular component, biological pathway, and molecular function are shown in blue, red, green, and purple, respectively. **(C)** Protein-protein interaction network was constructed with DEPs; no connective DEPs were excluded. The average ratios of DEPs are reflected by color intensity. Upregulated and downregulated DEPs are shown in red and green, respectively. Five DEPs included in the red ovals were further investigated by Western blotting analysis.

To comprehensively investigate the underlying mechanisms of PTC tumorigenicity with different degrees of LNMs, we obtained the global protein profiles of PTC tissues by performing TMT-based MS/MS. A total of 7,657 proteins were identified, of which 5,965 high confidence proteins without keratin were extracted with stringent criteria (q-value <0.01, unique peptide ≥2). The abundance of these 5,965 proteins was analyzed by non-supervised principal component analysis (PCA). Tumorous tissues in PTC-N0, PTC-N1a, and PTC-N1b were separated from pooled paired normal tissue (Npool_N) in Component 1, indicating that proteomic profiles in PTC tumorous tissues were distinct from paired normal tissues. Furthermore, tumorous tissues in PTC with different degrees of LNM also exhibited different profiles in terms of protein expression, resulting in a separate cluster in Component 2 (**Supplementary Figure 1B**). In further exploration of the patterns of variation among tumorous tissues in PTC-N0, PTC-N1a, and PTC-N1b, we obtained a total of 609 DEPs (q-value < 0.01, unique peptide ≥ 2, and fold change ≥ 1.7-fold or ≤ 0.4-fold) for hierarchical clustering analysis. The ratios of these 609 DEPs were grouped hierarchically into five clusters, of which 430 DEPs in cluster 5 were upregulated in N0_T, N1a_T, and N1b_T (**Figure 1A**). To further explore the biological significance of these DEPs, we performed GO and pathway enrichment analyses of these 430 DEPs. The majority of these DEPs were mainly involved in metabolism, energy pathways, cell growth, and extracellular matrix structural constituents (**Figure 1B**), which are the pathological hallmarks of cancer (21). Therefore, 70 DEPs enriched in these categories were extracted and average ratios were used to construct the PPI network. Of particular note, these DEPs were closely linked and upregulated in PTC tumorous tissues compared to the levels expressed in pooled normal tissues (**Figure 1C**).

### Verification of DEPs by Western Blotting and IHC Analyses

**Figure 2:**
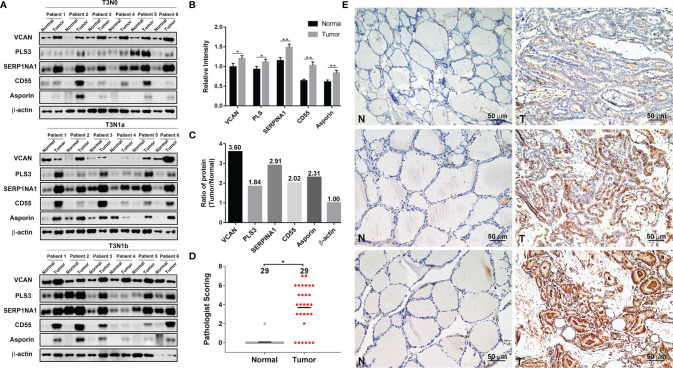
Validation of five differentially expressed proteins (DEPs) by Western blotting and IHC analyses. **(A)** VCAN, PLS3, SERP1NA1, CD55, and asporin protein levels were validated by Western blotting analysis; β-actin was used as the loading control. **(B)** Quantification of the indicated five DEPs relative to β-actin. **(C)** The average ratios of the indicated five DEPs were identified in the TMT-based proteomics data relative to β-actin. **(D)** Histopathological scoring of asporin in 29 paraffin-embedded PTC tumorous tissues and paired normal tissues. *P < 0.05, **P < 0.01. **(E)** Representative images of immunohistochemical labeling of asporin in PTC tumorous tissues and paired normal tissues. Asporin was expressed mainly in the extracellular matrix, cell membrane, and cytoplasm of PTC tumorous tissues, while a very low or no signal was detectable in paired normal tissues. Paired normal (N) or tumorous (T) tissues are marked with dotted lines. Scale bars, 50 μm.

To further validate our proteomics data, five core DEPs (VCAN, PLS3, SERP1NA1, CD55, and asporin) enriched in three different categories of PPI were analyzed by Western blotting (**Figure 2A**). VCAN, PLS3, SERP1NA1, CD55, and asporin were confirmed to be upregulated in in PTC-N0, PTC-N1a, and PTC-N1b tumorous tissues (**Figures 2A, B**); β-actin was used as an internal control. Thus, the Western blotting results were consistent with our proteomics data, and the average ratios of these five DEPs [e.g., Ratio_ave (asporin) = (N0_T/Npool_N + N1a_T/Npool_N + N1b_T/Npool_N)/3)] were 3.60, 1.84, 2.91, 2.02, and 2.31, respectively (**Figure 2C**). Asporin was selected for further investigation based on the following criteria: (a) Asporin acts as an oncogene in pancreatic, colorectal, gastric, and prostate cancer (10); (b) The roles of asporin in thyroid cancer have not been reported based in searchers of PubMed or Google. IHC performed in an independent set of PTC patients confirmed that the IHC scores of asporin were also increased in PTC tumorous tissues (**Figures 2D, E**). Furthermore, IHC staining indicated that asporin is expressed mainly in the extracellular matrix, cell membrane, and cytoplasm. Typical images of IHC staining of asporin expression are shown in **Figure 2E**. To further investigate the roles of asporin in PTC tumorigenesis, asporin RSEM data and corresponding clinicopathological parameters were successfully retrieved from the TCGA-THCA cohort. This dataset showed that the high-level Z-scores of asporin were positively correlated with PTC patients with larger tumor classification (*P* < 0.001), LNM (*P* < 0.001), high AJCC staging (*P* < 0.001), and *BRAF^V600E^* mutation (*P* < 0.001) (**Table 1**). Furthermore, we found that *asporin* mRNA expression showed a significant positive association with EMT-activating transcription factors (EMT-TFs), including *β-catenin* (r = 0.207, *P* < 0.001), *SLUG* (r = 0.706, *P* < 0.001), *ZEB1* (r = 0.428, *P* < 0.001), and *ZEB2* (r = 0.522, *P* < 0.001) (**Figure 4C**). These results suggested that asporin may exert a vital tumor-promoting function in PTC by regulating the MAPK/EMT axis.

**Table 1:** Correlations of *Asporin* mRNA expression in tumorous tissues with clinicopathological characteristics.

Characteristics	*Asporin* mRNA expression (Z scores)				
	*No.*	Low	Moderate	High	*P-*value
		(≤-1)	(-1 to 1)	(≥1)	
Age					
<45 years	221	30	165	26	0.29
≥45 years	269	35	181	53	
Sex					
Male	130	18	91	21	0.263
Female	360	47	255	58	
Multifocality					
Solitary	262	41	174	47	0.056
Multiple	218	22	164	32	
Tumor classification					
T1+T2	303	41	235	27	**<0.001**
T3+T4	185	24	109	52	
Lymph node classification					
N0	225	33	169	23	**<0.001**
N1	215	27	136	52	
AJCC Staging					
Stage I+II	324	44	250	30	**<0.001**
Stage III+IV	164	21	94	49	
*BRAF^V600E^* mutation					
Yes	231	24	156	51	**<0.001**
No	245	39	181	25	

P-value indicates the probability from the nonparametric Mann–Whitney test. Bold text indicates P < 0.05.

### Knockdown of Asporin Inhibits Cell Growth, Migration, and Invasion of Thyroid Cancer Cells

**Figure 3:**
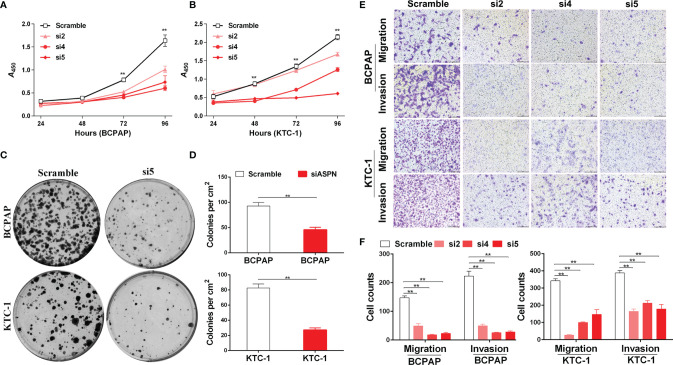
The knockdown of asporin inhibits thyroid cancer cell growth, migration, and invasion. **(A)** CCK-8 assay of the growth rates of control or siASPN BCPAP cells. Data represents the mean ± SD of three independent experiments. **P < 0.01(si4-si5 cultured for 72-96 h) **(B)** Growth rates of control or siASPN KTC-1 cells. **P < 0.01(si4-si5 cultured for 48-96 h,and si2 cultured for 96 h). **(C)** Representative images of colony formation in BCPAP and KTC-1 cells. **(D)** The number of clones counted in three independent experiments. Data are expressed as mean ± SD. **P < 0.01. **(E)** Representative images of Transwell assays of BCPAP and KTC-1 cell migration and invasion. **(F)** Cells that migrated across the chamber membrane were stained with 0.2% crystal violet solution and counted. Data represents the mean ± SD. **P < 0.01.

**Figure 4:**
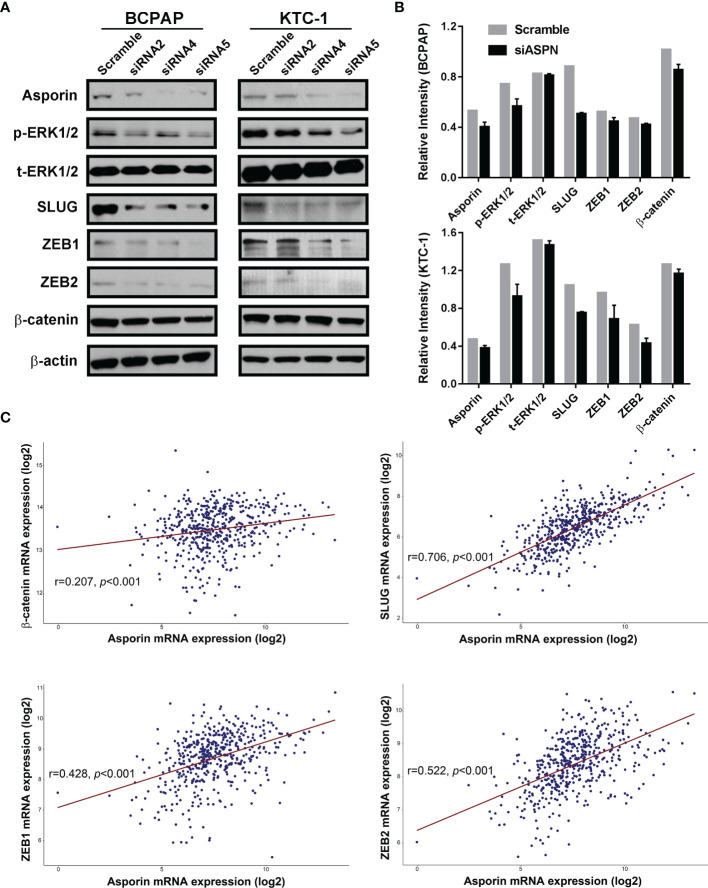
Asporin knockdown impairs the malignant phenotype of thyroid cancer cells by inhibiting the MAPK/EMT axis. **(A)** Knockdown of asporin using three siRNAs (siASPN). Levels of proteins in the MAPK signaling pathway and its downstream EMT-TFs were downregulated in the siASPN cells, including p-ERK1/2, SLUG, ZEB1, and ZEB2. The results presented are representative of at least 3 independent experiments. **(B)** Quantification of protein levels in control and siASPN cells relative to β-actin. **(C)** TCGA-THCA cohort data analysis indicated that asporin mRNA expression was positively correlated with the expression of some EMT-TFs mRNAs, including β-catenin, SLUG, ZEB1, and ZEB2.

To further examine the ability of asporin to enhance tumor progression in thyroid cancer, we knocked down endogenous *asporin* expression in thyroid cancer cell lines by transfection with three siRNAs (**Figures 4A, B**). Compared to the cells transfected with the scramble control, CCK-8 assays showed that *asporin* knockdown inhibited the viability of BCPAP and KTC-1 cells (**Figures 3A, B**). Furthermore, siASPN also decreased the number of colonies in the colony formation assays (**Figures 3C, D**), further indicating that *asporin* knockdown inhibits the growth of thyroid cancer cells. Next, we performed Transwell assays to examine the effects of *asporin* knockdown on the invasive and metastatic potential of these cells. We found that transfection with siASPN decreased the migratory and invasive ability of BCPAP and KTC-1 cells (**Figures 3E, F**). Interestingly, migration and invasion assays showed that siASPN also significantly decreased the ability of BHT101 cells (ATC cell line) to penetrate the Transwell membrane with or without Matrigel-coating (**Supplementary Figure 2**). These results suggested that *asporin* knockdown significantly inhibits the metastatic potential of thyroid cancer cells.

### Asporin Knockdown Impairs the Malignant Phenotype of Thyroid Cancer Cells by Inhibiting the MAPK/EMT Axis

Next, we investigated the molecular mechanism by which asporin promotes the malignant phenotype in thyroid cancer. TCGA-THCA cohort analysis indicated that *asporin* mRNA expression was positively associated with MAPK pathway activation and EMT-related mRNA expression. Western blotting analysis showed that asporin knockdown reduced p-ERK1/2 protein levels, but not t-ERK1/2 protein levels in BCPAP and KTC-1 cells (**Figures 4A, B**). Furthermore, siRNA-mediated silencing of asporin also resulted in the downregulation of EMT-TFs, including SLUG, ZEB1, and ZEB2, which is the downstream of the MAPK signaling pathway (**Figures 4A, B**). Of particular note, we also found that siRNA-mediated silencing of asporin resulted in the downregulation of p-ERK1/2, TWIST1, SLUG, ZEB1, and ZEB2 protein levels, and upregulation of E-cadherin in BTH101 cells (**Supplementary Figure 3**). These results indicated that asporin knockdown inhibited the tumorigenicity of thyroid cancer cells by hindering activation of the MAPK signaling pathway and downregulating its downstream EMT-TFs to impair the migration and invasiveness of thyroid cancer cells.

### Asporin Interacts With HER2 and Activates the HER2 Signaling Pathway

**Figure 5:**
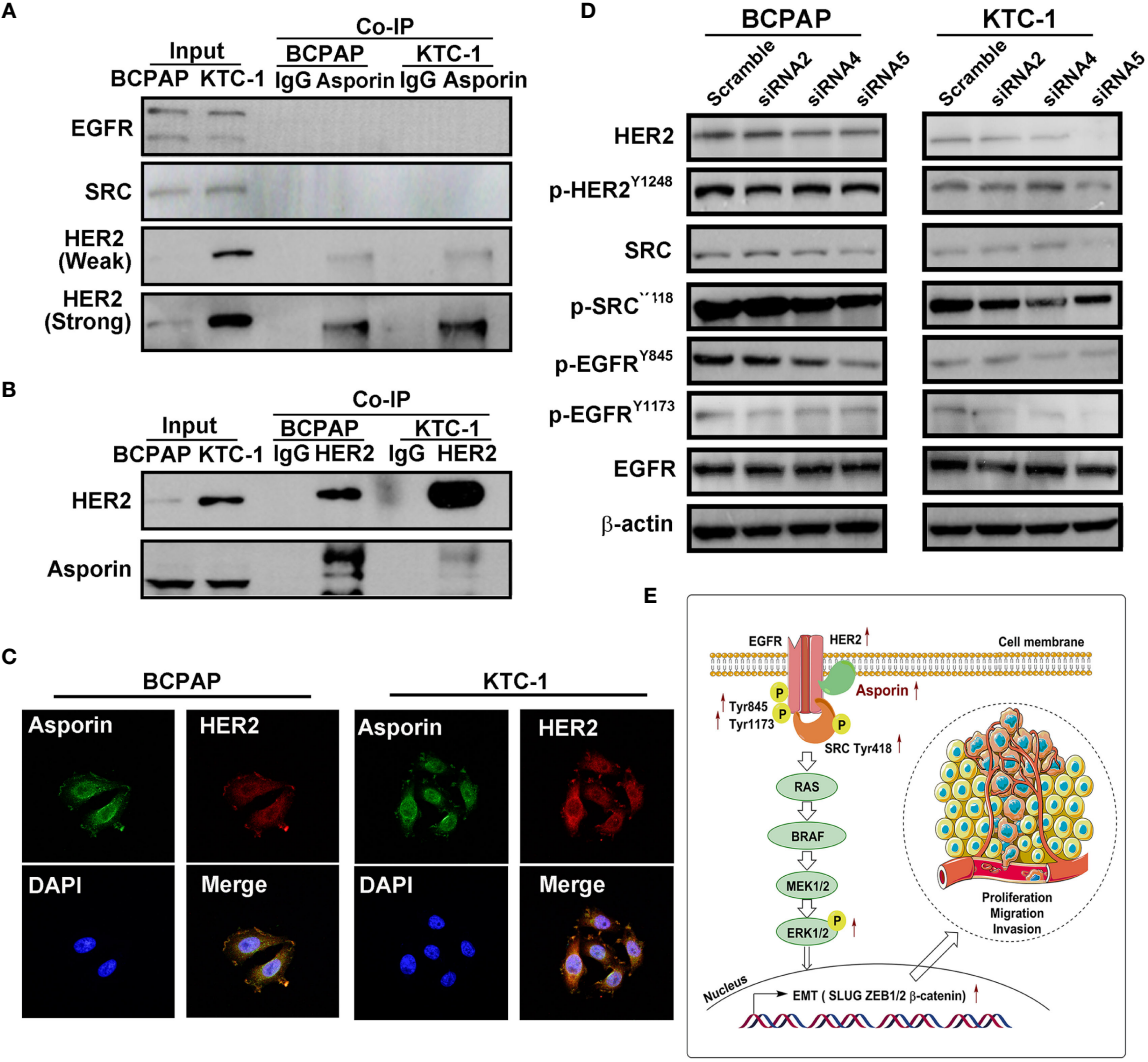
Asporin interacts with HER2 and activates the HER2 signaling pathway. **(A, B)** Endogenous co-immunoprecipitation (co-IP) assay revealed co-IP of asporin with HER2, but not with EGFR and SRC, and conversely, HER2 was coprecipitated with asprorin; IgG was used as the isotype control. The results presented are representative of at least 3 independent experiments. **(C)** Immunofluorescence staining of asporin (green) and HER2 (red) in BCPAP and KTC-1 cells. Merged images with DAPI staining. Asporin colocalizes with HER2 in the cell membrane and cytoplasm. One representative result of at least 3 independent experiments was shown. **(D)** Equal amounts of proteins in siASPN or control cells were analyzed by immunoblotting with the indicated antibodies. **(E)** Proposed working model for asporin promoting thyroid cancer metastasis by regulating the HER2/SRC/EGFR/MAPK/EMT axis.

Numerous studies have shown that the MAPK signaling pathway is activated by members of the EGF family, including EGFR and HER2 (22). We investigated the ability of asporin to interact with members of the EGF family in PTC using endogenous co-IP assays. Asporin was coprecipitated with HER2 but not with EGFR and SRC (**Figure 5A**), and conversely, HER2 was coprecipitated with asporin (**Figure 5B**). Furthermore, immunofluorescence assays revealed the co-localization of asporin and HER2 on the cell membrane and in the cytoplasm of BCPAP and KTC-1 cells (**Figure 5C**). These results indicated that asporin and HER2 form a complex. Knockdown of asporin expression in BCPAP and KTC-1 cell lines reduced HER2, p-HER2^Y1248^, p-SRC^Y418^, p-EGFR^Y845^, and p-EGFR^Y1173^ expression, but not SRC and EGFR levels (**Figures 5D, E**). These results suggested that asporin could bind HER2 to maintain its expression level, and asporin knockdown could subsequently downregulate the expression of p-EGFR and p-SRC in BCPAP and KTC-1 cell lines.

### Afatinib and PLX4032 Mimic the Effects of Asporin

**Figure 6:**
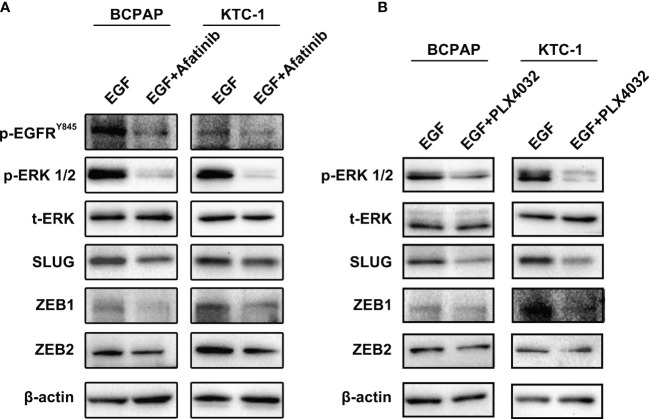
Afatinib and PLX4032 mimic the effects of Asporin knockdown on SLUG, ZEB1, and ZEB2, expression. **(A)** Afatinib treatment can downregulate p-EGFRY845, p-ERK1/2, SLUG, ZEB1, and ZEB2 expression, but not t-ERK1/2. **(B)** PLX4032 treatment results in the downregulation of p-ERK1/2, SLUG, ZEB1, and ZEB2 protein levels, but not t-ERK1/2.

Our results suggest that reduced MAPK pathway activity is due to lower EGFR/HER2 signaling which mediates the decrease in EMT regulating genes by Asporin knockdown. To confirm these results, pharmacological inhibitor assays were performed to confirm whether Afatinib (EGFR inhibitor) or PLX4032 (MAPK inhibitor) can mimic the effects of Asporin knockdown on SLUG, ZEB1, and ZEB2 expression. We found that BCPAP and KTC-1 cells treated with Afatinib can downregulate p-EGFR^Y845^, p-ERK1/2, SLUG, ZEB1, and ZEB2 expression, but not t-ERK1/2 (**Figure 6A**). Furthermore, PLX4032 treatment results in the downregulation of p-ERK1/2, SLUG, ZEB1, and ZEB2 protein levels in BCPAP and KTC-1 cells, but not t-ERK1/2 (**Figure 6B**).

### Clinical Applications of Asporin in PTC

**Figure 7:**
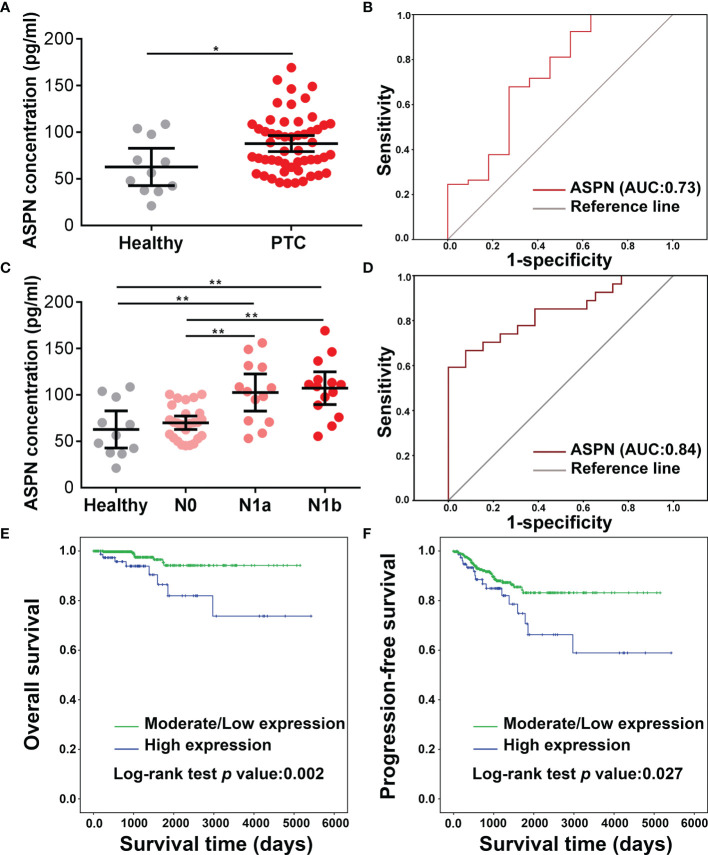
Clinical analysis of asporin expression in serum and tumorous tissues. **(A)** The serum levels of asporin in 54 PTC patients and 11 healthy volunteers were determined by sandwich ELISA. Data are expressed as mean ± SD. *P < 0.05, **P < 0.01. **(B)** Receiver-operator characteristic (ROC) curve analysis was used to examine the diagnostic efficacy of serum asporin levels. ROC curve analysis of data for discriminating PTC patients and healthy volunteers. **(C)** Serum asporin levels in patients with PTC-N0, PTC-N1a, and PTC-N1b. **(D)** ROC curve analysis of data for differentiating PTC-N0 from PTC-N1a and PTC-N1b.**(E, F)** Kaplan–Meier curves and log-rank tests showing high expression of asporin is positively correlated with worse progression-free survival and overall survival of patients with PTC.

Our *in vitro* results raised the possibility that the upregulation of asporin in serum represents a candidate biomarker in PTC. Accordingly, we examined the serological asporin levels in 54 PTC patients and 11 healthy volunteers by ELISA. The serum levels of asporin in PTC were higher than those in healthy volunteers (*P* < 0.05) (**Figure 7A**). We then used ROC curve analysis to determine the sensitivity and specificity of asporin as a biomarker in PTC. The area under the ROC curve (AUC) of asporin for discriminating PTC patients from healthy controls was 0.73, and the optimal Youden’s index was 0.407 (sensitivity = 0.679, specificity = 0.727) (**Figure 7B**). More importantly, serum levels of asporin in PTC-N1a and PTC-N1b patients were higher than those of healthy volunteers and PTC-N0 patients (*P* < 0.01) (**Figure 7C**). The AUC of asporin for discriminating PTC-N1a and PTC-N1b patients with PTC-N0 patients was 0.84, and the optimal Youden’s index was 0.59 (sensitivity = 0.667, specificity = 0.923) (**Figure 7D**). These results implicated asporin as a serological biomarker that can be used to identify PTC patients with or without lymph node metastasis. Furthermore, we referred to the TCGA-THCA cohort to investigate the potential correlation of asporin expression with OS or PFS. Kaplan–Meier analysis indicated that high Z-scores for asporin in tumorous tissues were associated with significantly worse PFS (*P* = 0.027) and OS (*P* = 0.002) than those of patients with normal/low Z-scores (**Figures 7E, F**). Therefore, elevated asporin expression in tumorous tissue was found to correlate positively with a poorer prognosis, thus, also implicating asporin as a novel candidate prognostic biomarker.

## Discussion

Our study revealed distinct tumorous protein profiles among PTC with different degrees of LNMs and showed that DEPs in tumorous tissues are mostly enriched in the extracellular matrix, metabolism, and cell growth. The proteomics data were validated by Western blotting analysis of VCAN, PLS3, SERP1NA1, CD55, and asporin, which were confirmed to be upregulated in PTC tumorous tissues with different degrees of LNMs. Asporin was found to be expressed mainly in the extracellular matrix, cell membrane, and cytoplasm of PTC tumorous tissues, and promoted thyroid cancer cell proliferation, migration, and invasion. Asporin was also shown to co-localize with HER2 on the cell membrane and in the cytoplasm of PTC cells. Furthermore, we showed that the asporin/HER2/SRC/EGFR axis upregulated the expression of EMT-TFs *via* the MAPK signaling pathway (**Figure 5E**). Finally, ELISA assay implicates asporin as a serological biomarker to identify PTC patients with or without lymph node metastasis, and high expression of asporin in PTC tumorous tissues is a risk factor for poor prognosis.

In colorectal cancer, Wu et al. demonstrated that asporin promoted cancer cell proliferation and metastasis *via* the EGFR/SRC/cortactin signaling pathway (23). Furthermore, Ding et al. suggested that asporin also promoted tumor growth and metastasis in gastric cancer *via* the EGFR/ERK/MMP2 axis (24). However, it is far from clear how asporin activates the EGFR signaling pathway to upregulate the p-EGFR protein level. Two previous studies suggested that HER2 and EGFR are overexpressed in PTC tumorous tissues (25, 26) and that HER2 and EGFR overexpression are positively associated with extrathyroidal extension, LNM, and high TNM stage in PTC (26). In the current study, we found that asporin interacted with HER2 and asporin knockdown downregulated protein levels of HER2, p-HER2^Y1248^, and p-EGFR^Y1171^. Mounting evidence shows that HER2 overexpression promotes EGFR expression and activity (27–29). Furthermore, HER2/HER2, HER2/EGFR, and HER2/HER3 levels were increased by HER2 overexpression, resulting in activation of the MAPK and PI3K signaling pathways, as well as stimulation of SRC kinases (30). In breast cancer, Jeong and colleagues found that PMCA2 knockdown disrupted the interaction between HER2 and HSP90 and promoted the internalization and degradation of HER2, resulting in a reduction in the protein levels of p-EGFR, HER3, and p-HER3, but not EGFR (31). Furthermore, Yoon et al. indicated that α6β4 integrin interacted with, and increased the translation of HER2 through eIF4E, which resulted in p-EGFR overexpression and activation of Ras to promote invasion in breast cancer cells (32). However, the mechanism by which asporin regulates HER2 expression in thyroid cancer remains to be fully elucidated. However, these previous studies provide good evidence that asporin may regulate HER2 expression at the translational or post-translational level.

A growing body of evidence indicates that SCR activity is necessary for HER2-mediated proliferation, survival, metastasis, and angiogenesis (33), suggesting that SRC is the vital second messenger of HER2. Furthermore, HER2 interacts with SRC to increase its expression and activity (34, 35). SRC also increases HER2/HER3 dimerization and HER2 activity (36), which indicates that HER2 and SRC may create a regulatory feedback loop. Interestingly, Biscardi et al. indicated that SRC also enhanced EGFR activity by inducing phosphorylation of Tyr845 and Tyr1101 (37). In accordance with previous studies, we also found that asporin knockdown in PTC cells downregulate protein levels of HER2, p-SRC^Y418^, and p-EGFR^Y845^, but not the total levels of SRC protein. Collectively, the asporin/HER2/MAPK/EMT axis promoted the migration and invasion of thyroid cancer cells.

Nearly 36% of PTC patients are diagnosed with LNMs, which are correlated with local tumor recurrence and cancer-specific mortality (38). Therefore, it is important to accurately diagnose the presence and level of LNMs. Although high-resolution ultrasound imaging can be used to evaluate the extent of primary tumors and LNMs of PTC (39), the overall sensitivity is only 51%, and this imaging has limitations for the evaluation of the deeply situated retropharyngeal and mediastinal lymph nodes (38, 39). In the present study, we found that serum asporin could not only be used to distinguish PTC patients from healthy volunteers but also to discriminate PTC-N1a and PTC-N1b patients from PTC-N0 patients. These results indicated that the combination of serum asporin levels and ultrasound imaging may be used to assess the probability that LNMs has occurred and the extent.

## Data Availability Statement

The datasets presented in this study can be found in online repositories. The names of the repository/repositories and accession number(s) can be found below:

https://www.ebi.ac.uk/pride/archive/, PXD007971.

## Ethics Statement

The studies involving human participants were reviewed and approved by Ethics Committee of Peking University Cancer Hospital & Institute. The patients/participants provided their written informed consent to participate in this study.

## Author Contributions

JML and WG conceived and designed the study. SZ, JL, HZ and TW performed all experiments. TW collected thyroid tissues and analyzed the primary data. SZ, JL and HZ drafted the manuscript. TW, JML. and WG proofread and revised the manuscript. All authors read and approved the final manuscript.

## Funding

This work was supported by the CAMS Innovation Fund for Medical Sciences (CIFMS #2017-I2M-3-001), the National Natural Science Foundation of China (No. 82103111 and 81971023), and Key Clinical Projects of Peking University Third Hospital (BYSYZD2021035).

## Conflict of Interest

The authors declare that the research was conducted in the absence of any commercial or financial relationships that could be construed as a potential conflict of interest.

## Publisher’s Note

All claims expressed in this article are solely those of the authors and do not necessarily represent those of their affiliated organizations, or those of the publisher, the editors and the reviewers. Any product that may be evaluated in this article, or claim that may be made by its manufacturer, is not guaranteed or endorsed by the publisher.
