# Supplementary material for: Asporin Interacts With HER2 to Promote Thyroid Cancer Metastasis via the MAPK/EMT Signaling Pathway
Source: Front Oncol. 2022 May 2;12:762180. doi: 10.3389/fonc.2022.762180 (PMC9119632; doi:10.3389/fonc.2022.762180)
Supplement: Supplementary file 1 [file DataSheet_1.docx]

**Supplementary information**

**
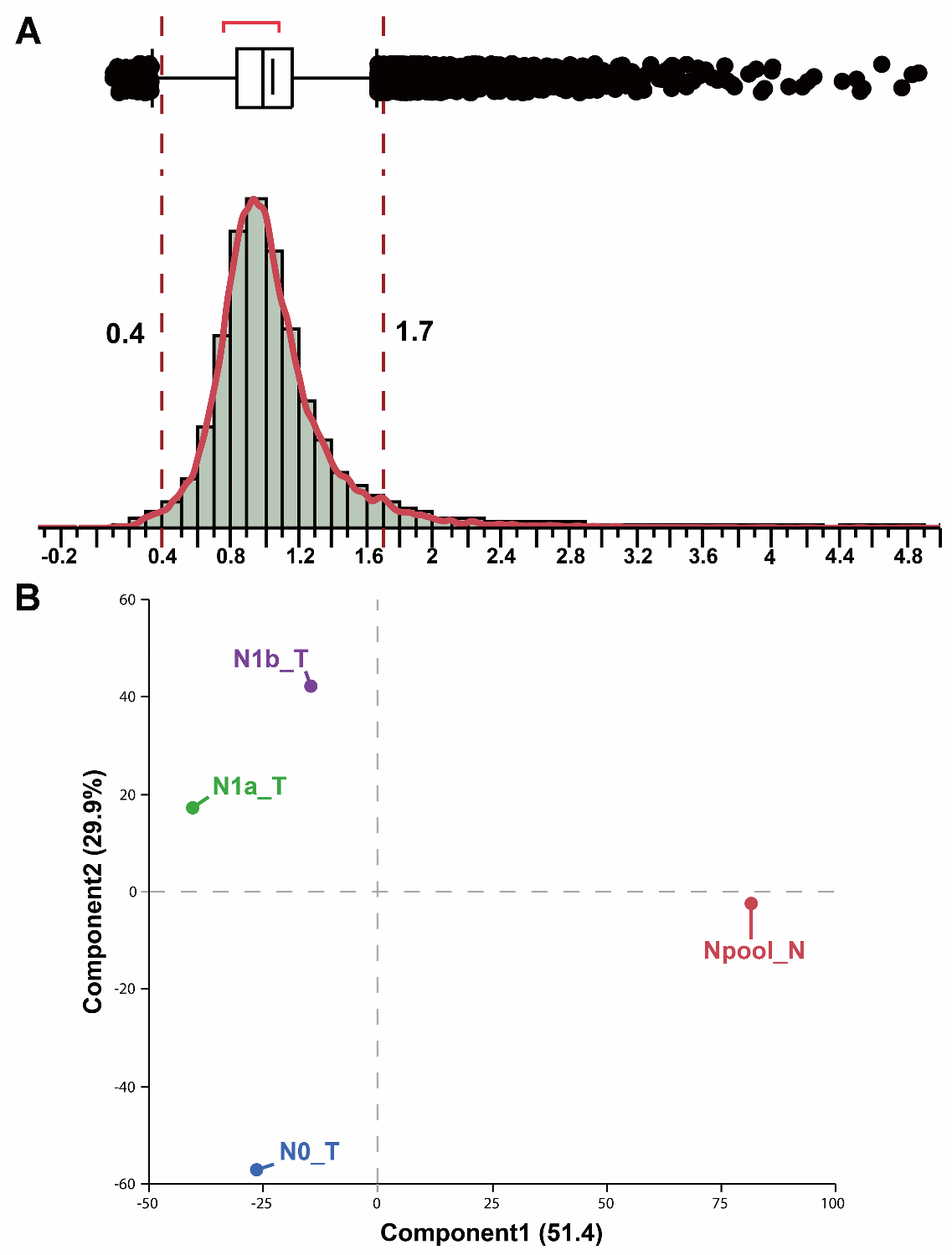
**

**Supplementary Figure.1**

Bioinformatic analysis of differentially expressed proteins (DEPs) by TMT-based MS/MS. (A) The combined ratio distribution of proteins calculated to identify the cutoffs for DEPs (set as ≥ 1.7-fold or ≤ 0.4-fold). (B) Non-supervised PCA analysis.


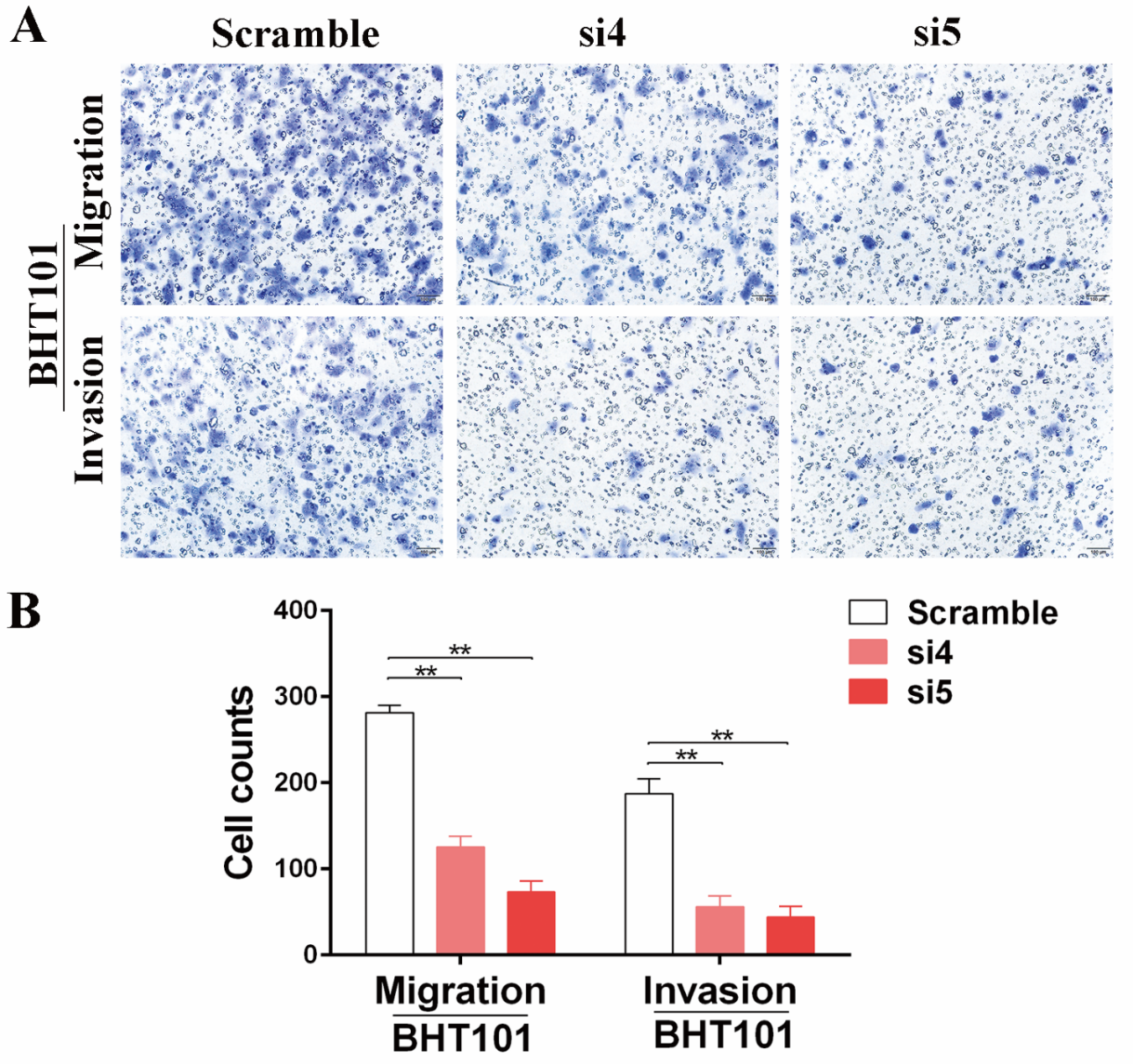


**Supplementary Figure.2**

Knockdown of asporin inhibits cell migration and invasion of ATC cells. (A) Representative images of Transwell assays of BHT101 cell migration and invasion. (B) Cells that migrated across the chamber membrane were stained with 0.2% crystal violet solution and counted. Data represent the mean ± SD. ** *P* < 0.01.


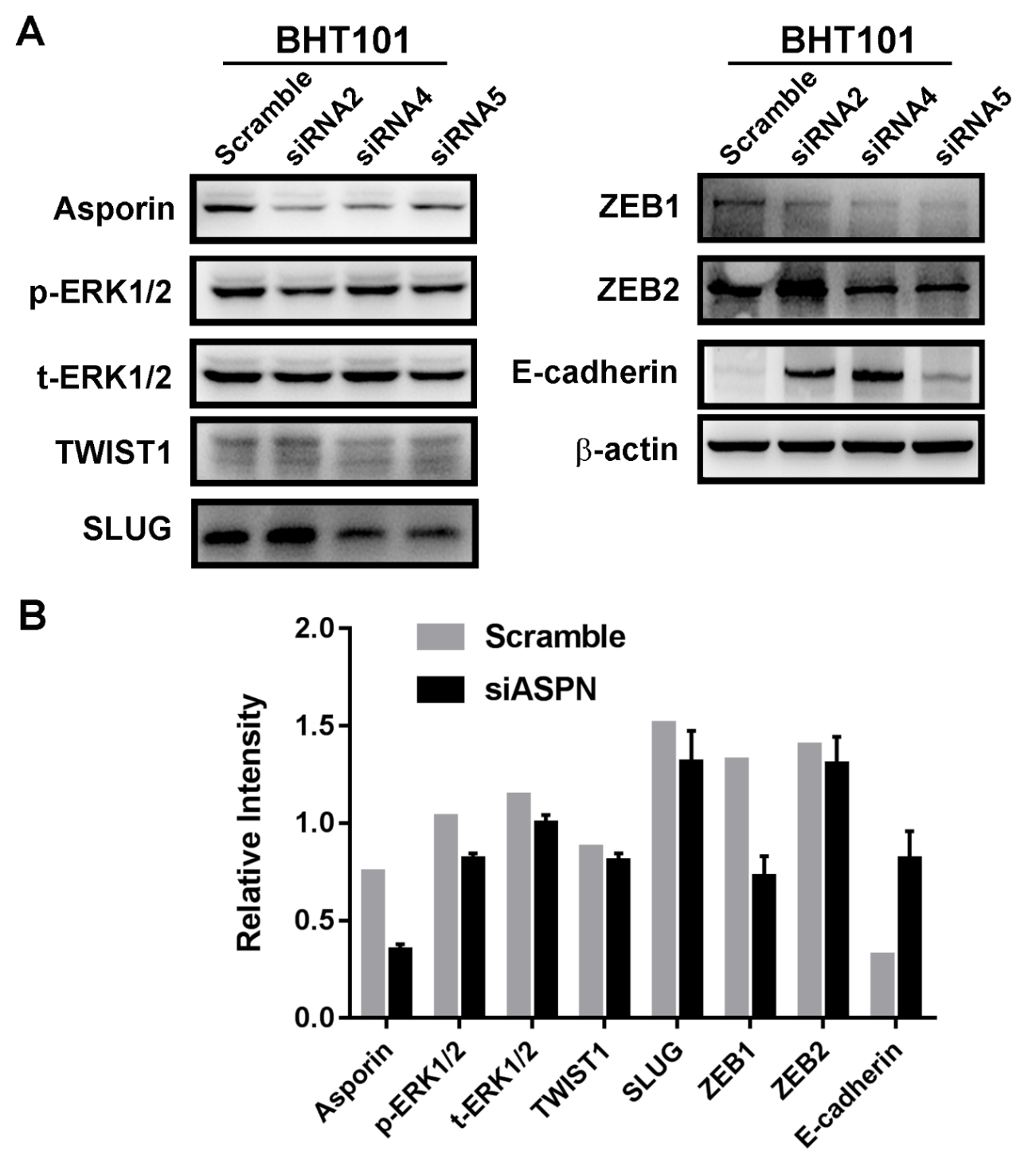


**Supplementary Figure.3**

Asporin knockdown impairs the malignant phenotype of ATC cells by inhibiting the MAPK/EMT axis. (A) Knockdown of asporin using three siRNA (siASPN). Equal amounts of proteins in siASPN or control cells were analyzed by immunoblotting with the indicated antibodies. (B) Quantification of the indicated proteins relative to β-actin.

**Supplementary Table 1.** Detailed information of PTC patients in TMT-based MS/MS, IHC, and ELISA assays.
